# Anisotropic finite element models for brain injury prediction: the sensitivity of axonal strain to white matter tract inter-subject variability

**DOI:** 10.1007/s10237-017-0887-5

**Published:** 2017-02-23

**Authors:** Chiara Giordano, Stefano Zappalà, Svein Kleiven

**Affiliations:** Royal Institute of Technology KTH, School of Technology and Health, Hälsovägen 11C, 141 57 Huddinge, Sweden

**Keywords:** Axonal strain, Brain anisotropy, Traumatic brain injury, Finite element analysis

## Abstract

Computational models incorporating anisotropic features of brain tissue have become a valuable tool for studying the occurrence of traumatic brain injury. The tissue deformation in the direction of white matter tracts (axonal strain) was repeatedly shown to be an appropriate mechanical parameter to predict injury. However, when assessing the reliability of axonal strain to predict injury in a population, it is important to consider the predictor sensitivity to the biological inter-subject variability of the human brain. The present study investigated the axonal strain response of 485 white matter subject-specific anisotropic finite element models of the head subjected to the same loading conditions. It was observed that the biological variability affected the orientation of the preferential directions (coefficient of variation of 39.41% for the elevation angle—coefficient of variation of 29.31% for the azimuth angle) and the determination of the mechanical fiber alignment parameter in the model (gray matter volume 55.55–70.75%). The magnitude of the maximum axonal strain showed coefficients of variation of 11.91%. On the contrary, the localization of the maximum axonal strain was consistent: the peak of strain was typically located in a 2 cm^3^ volume of the brain. For a sport concussive event, the predictor was capable of discerning between non-injurious and concussed populations in several areas of the brain. It was concluded that, despite its sensitivity to biological variability, axonal strain is an appropriate mechanical parameter to predict traumatic brain injury.

## Introduction

Traumatic brain injury (TBI) occurs when a sudden trauma causes the brain to malfunction. The injury is generally produced by a bump, a blow, or jolt to the head or the penetration of the tissue by an object. Depending of the extent of the damage, symptoms of TBI may range from a brief change in mental status or consciousness (mild TBI) to an extended period of unconsciousness, amnesia, or even death (severe TBI).

High rates of deficit and morbidity are unfortunately associated with TBI. In the United States, between 2001 and 2006, data compiled by the Centers for Disease Control and Prevention indicated that each year on average 1.7 million people suffered an injury and sought medical help. The treatments consisted of 80.7% emergency department visits, 16.3% hospitalizations and 3% deaths. The total estimated direct and indirect medical cost was 76.5 billion dollars (Faul et al. 2010). In Europe, year 2006, Tagliaferri et al. (2006) reported a TBI incidence rate of about 235 per 100,000 and an average mortality rate of about 15 per 100,000. Olesen et al. (2012) calculated that the total European 2010 cost of TBI (direct health care, direct non-medical, and indirect) was around 33 billion euros.

The diagnosis of TBI is controversial. Impairment of cognitive skills, such as thinking and language, and alterations in perception and emotions are not easily quantifiable. Furthermore, currently available neuroimaging techniques do not capture brain tissue damage, especially at the brain smallest functional unit, the neuron (Shenton et al. 2012). Consequently, the mechanisms underlying cell death are not fully understood yet but are under continuous investigation. One of the most realistic mechanisms of TBI seems to be the deformation of the axons. Gennarelli et al. (1998) suggested that, if the axon is stretched, ionic perturbations occur to maintain osmotic balance in the neuron. This causes local swelling and impairment of axonal transport. In following years, many studies confirmed that tissue deformation relates to injury (Bain et al. 2001; Shaw 2002; Kang and Morrison 2015).

To investigate the relationship between mechanical forces developed during an impact and the resulting brain injury, finite element (FE) models of the human head have been increasingly used. These mathematical models, which combine an accurate representation of the head anatomy and sophisticated material properties, have the potential to simulate tissue loads and deformation patterns of brain structures. Local mechanical parameters derived from strain and stress tensors can be extracted and used as injury predictors (Zhang et al. 2004; Mao et al. 2006; Kleiven 2007; Takhounts et al. 2008; Giordano and Kleiven 2014). Moreover, in vitro research showed that the occurrence of TBI is related to both viscoelastic properties and highly organized structure of the brain tissue (Bain and Meaney 2000; Smith and Meaney 2000; Bain et al. 2001; Elkin and Morrison 2007). Thus, in order to accurately emulate brain mechanical behavior, FE models need to incorporate anisotropic features of the brain and consider the deformation in the specific white matter tract direction.

Information of white matter tract orientation can be obtained from diffusion tensor images (DTIs) (Mori and Zhang 2006). In the last years, few studies utilized diffusion information at different levels to incorporate the effects of inhomogeneity and anisotropy of brain tissue into injury analysis (Colgan et al. 2010; Chatelin et al. 2011; Wright and Ramesh 2012; Kraft et al. 2012; Giordano and Kleiven 2014; Sahoo et al. 2015; Ji et al. 2015). In particular, in a study by Giordano and Kleiven (2014), an anisotropic FE model of the human head was developed and the brain tissue was modeled as a hyper-viscoelastic fiber-reinforced material. The orientation of white matter tracts was derived element-wise from DTIs and the deformation in their direction (axonal strain) was proposed as injury criterion. For a data set of mild TBI from the American football league, the strain in the axonal direction was found to be the best injury predictor. This result was confirmed in a study by Sahoo et al. (2015) where another anisotropic FE model was used for numerical computation of 109 head traumas. The authors showed that axonal strain was the appropriate candidate parameter to predict diffuse axonal injury.

A limitation of the FE models by Giordano and Kleiven (2014) and Sahoo et al. (2015) was that the anisotropic properties of the brain were extracted from diffusion data of a single healthy subject (Giordano et al. 2014) or from average diffusion data of 12 healthy subjects (Sahoo et al. 2015). However, several studies proved that the orientation of white matter tracts vary substantially in a population (Bürgel et al. 1999; Bürgel and Amunts 2006; Veenith et al. 2013; Sadeghi et al. 2015). For example, Bürgel and Amunts (2006) showed that a significant inter-subject variability exists for each tract within the single hemispheres and Veenith et al. (2013) also found a high coeffiecient of variability (5–7%) in measurements obtained from different subjects. Since the mechanical properties of an anisotropic FE model are strictly dependent on the orientation of the preferential directions, it follows that anisotropic FE models of the human head based on different subjects will likely respond differently to injurious loads, affecting their capability of predicting TBI. On top of the modeling uncertainties due to the approximation of the geometry and material properties, the FE calculations will be affected by uncertainties in the determination of the correct alignment of the white matter tracts. In particular, when anisotropic predictors are promoted (such as maximum axonal strain), the modelers should be careful to investigate the uncertainty of the predictions due to biological variation. However, to the best knowledge of the authors, an investigation of this effect has never been performed.

The aim of this study was therefore to investigate the uncertainty of TBI predictions of an anisotropic FE model due to white matter tract variability. The same loading scenario was imposed on 485 subject-specific FE head models where the orientation of white matter tracts was determined from the subject’s DTI. This investigation helped determining wheter the uncertainty introduced by the orientation of white matter tracts was influencing the capability of the axonal strain to separate injured and healthy populations.

**Fig. 1:**
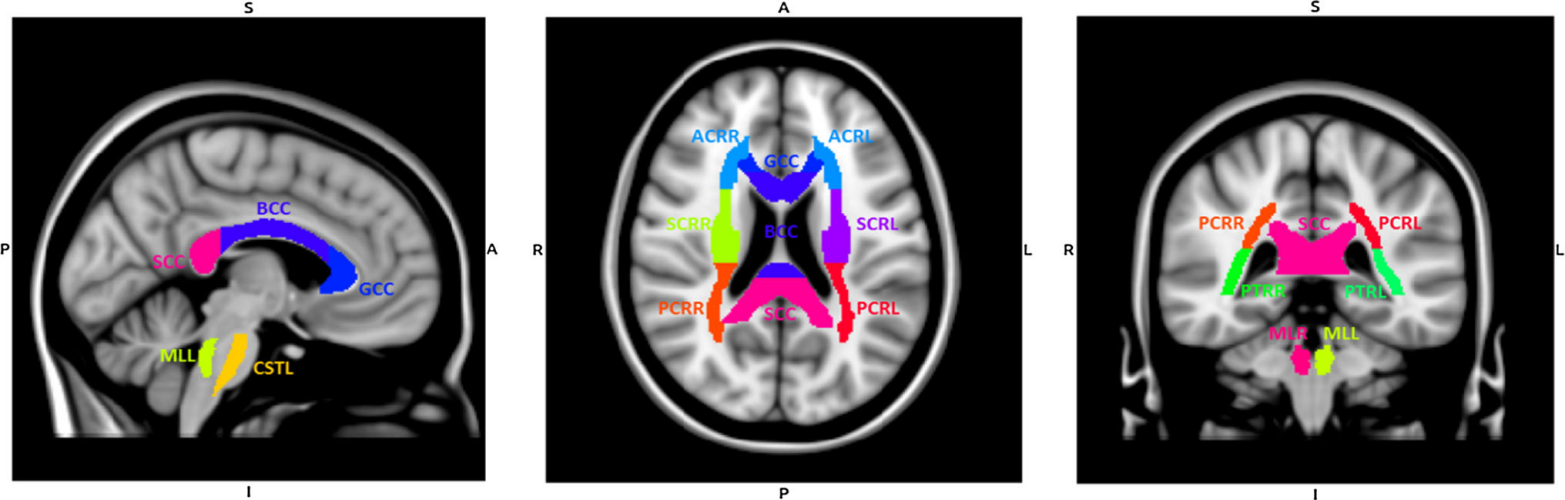
Sagittal (*left*), coronal (*middle*) and transversal (*right*) sections at $x=-1$, $y=-17$ and $z=19$ of the T1w MNI152 template. The regions of interest for TBI are labeled in color (ICBM-DTI-81 white matter tractography atlas). Labeling: *GCC* genus of corpus callosum, *BCC* body of corpus callosum, *SCC* splenium of corpus callosum, *CST* cortico-spinal tract, *ML* medial lemniscus, *ACR* anterior corona radiata, *SCR* superior corona radiata, *PCR* posterior corona radiata, *PTR* posterior thalamic radiation. *L* and *R* stands for *left* and *right*

## Method

### Human connectome dataset

The data used in this study were publicly released by the WU-Minn Human Connectome Project (HCP) consortium in the 500 Subjects release (June 2014) and the MEG2 Release (November 2014, Open Access). The HCP subjects were drawn from a population of adult twins and their non-twin siblings, in the age range of 22–35 years. The HCP aimed at characterizing the structure of the major brain pathways, understanding essential brain circuits and get insights into the functions that depend on them (Sotiropoulos et al. 2013). Among the different types of images included in the dataset, 485 diffusion weighted images (DWIs) were used in this study.

High angular diffusion images generally consisted of 288 volumes with an isotropic resolution of 1.25 mm. The *b* vectors and the *b* values were available in a text file, as well as a binary volume mask representing the brain area where to limit diffusion calculations. After the acquisition, diffusion images were processed with a pipeline including normalization of the intensities across runs and correction for EPI distortion, eddy current, motion artifacts, and gradient nonlinearities. Moreover, the DWIs were registered to the T1w native space, in order to have the structural and diffusion images defined in the same subject-specific space. More details regarding the dataset are reported in the reference manual (HCP 2014).

### Diffusion tensor estimation, registration, and tractography

The estimation of the diffusion tensors and the extraction of the eigenvalue and eigenvector maps were implemented through the DTIFIT tool of the diffusion toolbox (FDT) in FSL (Oxford Centre for Functional MRI of the Brain, FMRIB). For each subject, a weighted linear least squares procedure was applied to the diffusion weighted images acquired with *b* values smaller than 1500 s/mm^2^. The selection of these specific gradients was done to obtain the best mono-exponential fit (Kristoffersen 2011). The effect of the gradient nonlinearity on the *b* vectors was also considered (Sotiropoulos et al. 2013). According to Koay et al. (2006), negative eigenvalues were set to zero to guarantee the positive definitiveness of the diffusion tensors. The iteration over the 485 subjects was automated through a script written in Bash command language and executed in the Unix Shell.

Successively, in order to compare data acquired from different subjects, all the diffusion tensor images were registered to the Montreal Neurological Institute (MNI) space (Grabner et al. 2006) through nonlinear registration (scaling, shearing and warping). The alignment to a common template was automated over the 485 subjects through a script written in Bash command language and executed in the Unix Shell. In particular, the Functional MRI Software Library v5.0 in FSL and the package for nonlinear registration, FNIRT, were used. The warping maps available for each subject in the HCP dataset (Glasser et al. 2013) were given in input to the FNIRT toolbox. During the calculations, the principal direction of diffusion was preserved (Alexander et al. 2001).

Once all the images were aligned in the MNI space, the ICBM-DTI-81 white matter tractography atlas (Mori et al. 2008) was used to identify regions of interest (ROIs) and divide different white matter tracts during the analysis. This atlas was indeed defined in the MNI space and contained 48 labels identifying the major white matter tracts in the brain. In this study, 15 labels were used for the analysis. Important regions of interest for TBI were the cortico-spinal tracts (CST) and the medial lemniscus (ML) in the brainstem, the commissural fibers in the genus, body and splenium of the corpus callosum (GCC, BCC, SCC), anterior, superior, and posterior corona radiata (ACR, SCR, PCR) and the posterior thalamic radiation (PTR). Figure 1 illustrates their location in the MNI space.

Deterministic tractography was executed in UCL Camino Diffusion MRI Toolkit. The tracking algorithm was based on a fourth order Runge-Kutta method (Basser et al. 2000). In particular, fibers were seeded in the voxels of the 15 regions corresponding to the ICBM-DTI-81 atlas. To exclude boundary fibers and reduce the impact of misregistration, the ROI template was eroded by a voxel, as reported in Veenith et al. (2013). The construction of the fibers was stopped either in correspondence of voxels with fractional anisotropy lower than 0.2 or when the curve direction changed more than 40 degrees in 1 mm. The step size of the fibers was fixed to be 0.5 mm where new points were linearly interpolated.

**Fig. 2:**
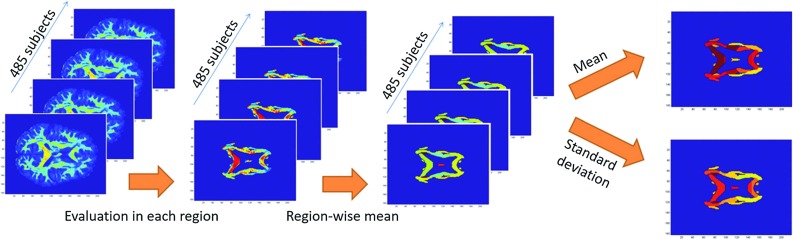
Schematic representation of the statistical analysis of the fractional anisotropy

**Fig. 3:**
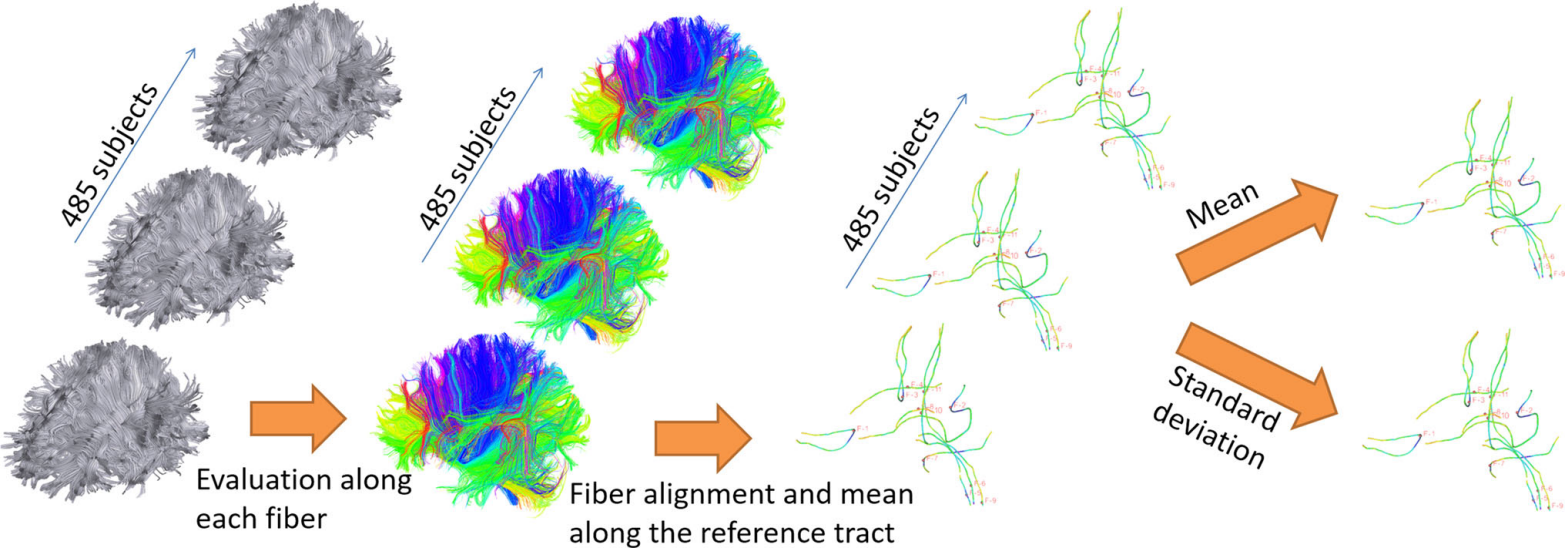
Schematic representation of the procedure for evaluating the fiber shape descriptors

### Scalar parametric maps

From the previously estimated diffusion tensor images (DTIs) scalar parametric maps (Mori and Zhang 2006) were extracted in order to study the microstructural organization of the brain tissue in a healthy population and define mechanical parameters for patient specific FE models. A MATLAB script estimated the fractional anisotropy (FA) map as following:

1 $$\begin{aligned} \mathrm{FA}=\root \of {\frac{(\lambda _1 - \lambda _2)^2+(\lambda _2-\lambda _3)^2+(\lambda _3-\lambda _1)^2}{2(\lambda _1^2+\lambda _2^2+\lambda _3^2)}} \end{aligned}$$

with $\lambda _i$ eigenvalues of the diffusion tensor (**DiffT**).

For each subject, the mean value of the diffusion parameter was calculated in each of the 15 ROIs. Successively, the mean and the standard deviation of previously extracted values were estimated among different subjects. The rationale for the fractional anisotropy analysis is reported in Fig. 2.

### Statistical analysis of white matter tracts

The previously estimated white matter tracts (tractography) were analyzed in terms of length, curvature, and orientation in the three-dimensional space. The analysis was performed for each of the 485 subject in the 15 ROIs using a MATLAB script. A schematic representation of the analysis is reported in Fig. 3.

The length of a fiber was defined as the product of the step size $\varDelta m$ (fixed to 0.5 mm) and the number of points forming the fiber, *n*, as below:

2 $$\begin{aligned} l=\varDelta m (n-1) \end{aligned}$$

Each fiber was geometrically characterized with local shape descriptors (Gerig et al. 2004; Corouge et al. 2004). The geometrical characteristics were derived from the Frenet frame, a non-inertial coordinate system attached to each point of the fiber. To identify the local orientation of a fiber in the three-dimensional space, the unit tangent vector **T**(s) was extracted at each point forming the fiber according to:

3 $$\begin{aligned} \mathbf T (s)=\frac{\frac{\mathrm{d}{} \mathbf r (s)}{\mathrm{d}s}}{\left\| \frac{\mathrm{d}{} \mathbf r (s)}{\mathrm{d}s}\right\| } \end{aligned}$$

**Fig. 4:**
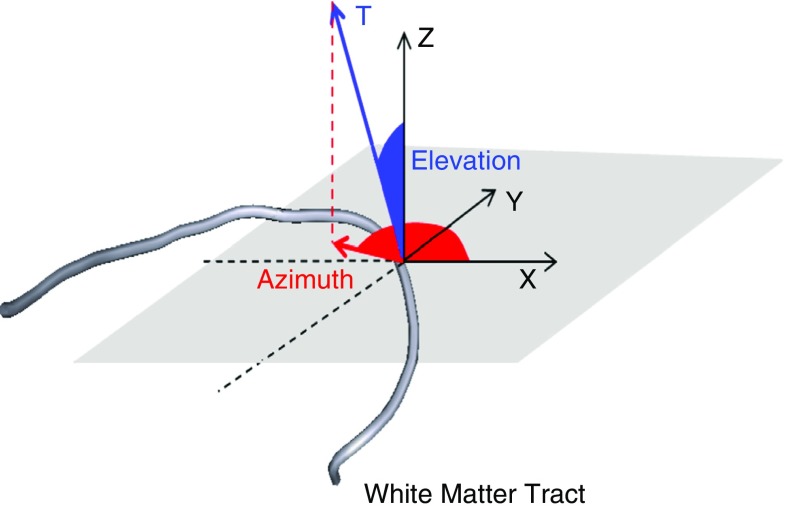
Characterization of the fiber tangent vector **T** by means of $\varTheta \in [0,\pi ]$, elevation angle, and $\varPhi \in [0,2\pi ]$, azimuth angle, in a three-dimensional Cartesian coordinate system $\{\mathbf{e }_1,\mathbf{e }_2,\mathbf{e }_3\}$

where $\mathbf r (s)$ represented the fiber tract parameterized by the arc length *s*. The fiber orientation was expressed in terms of two Eulerian angles $\varTheta \in [0,\pi ]$ (elevation angle) and $\varPhi \in [0,2\pi ]$ (azimuth angle) in a three-dimensional Cartesian coordinate system $\{\mathbf{e }_1,\mathbf{e }_2,\mathbf{e }_3\}$ (Fig. 4).

4 $$\begin{aligned} \mathbf T (\varTheta , \varPhi )=\mathrm{sin}(\varTheta )\mathrm{cos}(\varPhi )\mathbf e _1 + \mathrm{sin}(\varTheta )\mathrm{sin}(\varPhi )\mathbf e _2 + \mathrm{cos}(\varTheta )\mathbf e _3 \end{aligned}$$

Finally, the local curvature of a white matter tract was computed as the failure of a curve to be a straight line:

5 $$\begin{aligned} k(s)=\left\| \frac{\mathrm{d}{} \mathbf T (s)}{\mathrm{d}s}\right\| \end{aligned}$$

Only fibers with a minimum length of 10 mm were analyzed, in order to exclude tracts that terminated prematurely. The threshold value was selected from the histogram of the lengths in order to include the majority of the tracts.

**Fig. 5:**
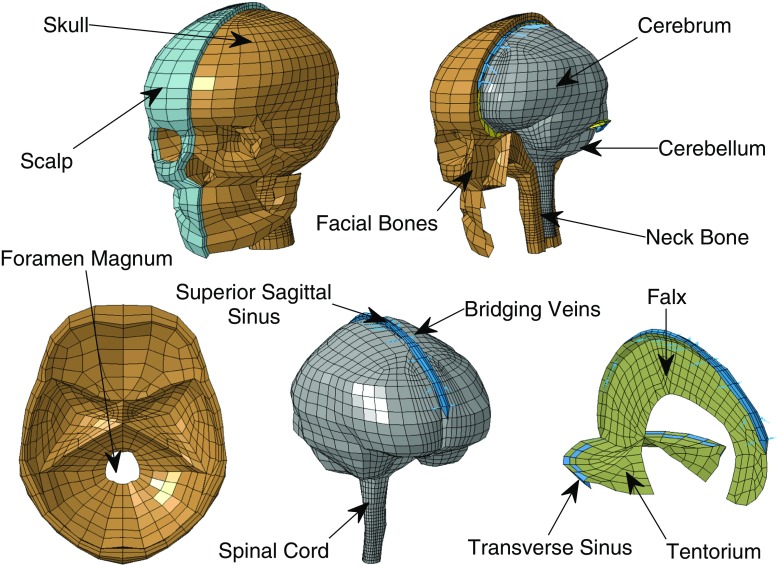
Baseline FE model of the human head (Giordano and Kleiven 2014). *On the top* isometric view of the head model with open scalp and exposed skull. Internal view of the head model with open skull and exposed brain. *On the bottom* details of the skull base, brain membranes and bridging veins

**Table 1:** A summary of the material properties of the head model components used in this study

Tissue	Young’s modulus (MPa)	Density (Kg/dm^3^)	Poisson’s ratio
Outer compact bone	15,000	2.00	0.22
Inner compact bone	15,000	2.00	0.22
Porous bone	1000	1.30	0.24
Neck bone	1000	1.30	0.24
Cerebrospinal fluid	$K=2.1$ GPa	1.00	–
Brain	Hyper-viscoelastic fiber-reinforced GOH (see Table 3)		
Sinuses	$K=2.1$ GPa	1.00	–
Dura mater	31.5	1.13	0.45
Falx	31.5	1.13	0.45
Tentorium	31.5	1.13	0.45
Pia mater	11.5	1.13	0.45
Scalp	Viscoelastic	1.13	0.42
Bridging veins	EA $= 1.9$ N	–	–

The capital letter *K* represents the Bulk Modulus while EA means force/unit strain. The brain is assigned a hyper-viscoelastic fiber-reinforced anisotropic material

To compare the orientation and curvature of the tracts in a specific region of interest, the reference fiber was identified as the one passing through the center of the ROI. The other tracts were then aligned by making their seed point corresponding to the closest point of the reference. The search of the closest point was only executed in 12 mm before and after the reference seed point in order to find the best correspondence in the ROI’s area. After the alignment the mean and standard deviation of **T**(s) and *k*(s) for each arch-length value were calculated. These quantities represented the evolution of the average tangent vector and the average curvature along the reference fiber in a particular ROI. From these mean ROI values, the mean and the standard deviation of the average length and the average evolution of **T**(s) and *k*(s) were calculated among the 485 subject.

### Generation of white matter subject-specific FE models

The FE models used in this study were white matter subject-specific adaptations of the anisotropic FE model developed by Giordano and Kleiven (2014). The baseline model (Giordano and Kleiven 2014) consisted of 16,906 nodes, 11,158 eight node brick elements, 10,165 four-node shell elements and 22 two-node truss elements. As it can be seen in Fig. 5, it included the scalp, the skull, the brain, the meninges, the CSF, the ventricles, 11 of the largest parasagittal bridging veins and a simple neck with the extension of the spinal cord and the dura mater. Details about model validation are reported in “Appendix 1.” Further detail can be found in the publication by Giordano et al. (2014), Giordano and Kleiven (2014), Giordano and Kleiven (2016). Tables 1, 2 and 3 describe the material models used in the simulations. The brain was assigned a Gasser–Ogden–Holzapfel (GOH) hyper-viscoelastic fiber-reinforced anisotropic law defined by the hyper-elastic strain energy potential:

6 $$\begin{aligned} \begin{aligned} W&=\frac{G}{2}(\tilde{I}_1 - 3) + K \left( \frac{J^2-1}{4} - \frac{1}{2} \mathrm{ln}(J)\right) \\&\quad +\frac{k_1}{k_2}(e^{k_2 \langle \tilde{E}_a^2 \rangle }-1)\\ \end{aligned} \end{aligned}$$

where

7 $$\begin{aligned} \begin{aligned} \tilde{E}_a&= k(\tilde{I}_1 - 3) +(1-3k)(\tilde{I}_{4\alpha })\\ \tilde{I}_{4\alpha }&= \tilde{\mathbf{C }} : \mathbf {n_{0\alpha }} \otimes \mathbf {n_{0\alpha }} \\ \end{aligned} \end{aligned}$$

In the expression *W* represents the strain energy per unit of reference volume, *G* and *K* are the shear and the bulk modulus respectively, *J* is equal to the determinant of the deformation gradient, $\tilde{I}_1$ corresponds to the first invariant of the isochoric Cauchy–Green strain tensor, and $k_1$ and $k_2$ describe the fiber stiffness (Gasser et al. 2006). The gray matter behavior was assumed to be isotropic ($k=0.3333$), while the degree of anisotropy of the white matter was discretized into intervals based on FA (Table 2).

In order to generate white matter subject-specific FE models, the diffusion information of a single subject was mapped within the geometry of the baseline model. The protocol proposed by Giordano et al. (2014) was followed: as first the FE brain mesh was voxelized to a reference volume; subsequently the DTI brain and the voxelized brain mask were aligned by affine registration. Finally the DTI volume was transformed to the FE reference using the preservation of principal direction algorithm (FSL). To insert the diffusion information into the FE model, all DTI voxels belonging to a single finite element of the brain were identified based on spatial coordinates. Diffusion information was averaged to extract the mean element anisotropy information according to

8 $$\begin{aligned} \mathbf DiffT _\mathrm{element}=\frac{\sum _{i=1}^{N} \mathbf DiffT _i \cdot {\text {e}}^{-D_i}}{\sum _{i=1}^{N} {\text {e}}^{-D_i}} \end{aligned}$$

**Table 2:** Discretization of fractional anisotropy in intervals and respective *k* values for FE simulations (Giordano et al. 2014)

FA range	*k* value
0.0–0.2	0.3333
0.2–0.3	0.2732
0.3–0.4	0.2500
0.4–0.5	0.2273
0.5–0.6	0.2000
0.6–0.7	0.1667
0.7–0.8	0.1282
0.8–0.9	0.0769
0.9–1.0	0.0000

**Table 3:** Properties of brain tissue in the head model: parameters refer to the hyper-viscoelastic fiber-reinforced anisotropic formulation (Giordano and Kleiven 2014)

Parameter	Value
*G* (Pa)	2990
*K* (MPa)	50
*k*	Depending on FA
$k_1$	Depending on location
$k_2$	$\rightarrow$ 0
$M_1\; \mathrm{for} \ \tau _1 = 10^{-6}\,\mathrm{s}$	0.7685
$M_2\; \mathrm{for} \ \tau _2 = 10^{-5}\,\mathrm{s}$	0.1856
$M_3\; \mathrm{for} \ \tau _3 = 10^{-4}\,\mathrm{s}$	0.0148
$M_4\; \mathrm{for} \ \tau _4 = 10^{-3}\,\mathrm{s}$	0.0190
$M_5\; \mathrm{for} \ \tau _5 = 10^{-2}\,\mathrm{s}$	0.0026
$M_6\; \mathrm{for} \ \tau _6 = 10^{-1}\,\mathrm{s}$	0.0070
$M_{\infty }$	0.0025

Viscoelasticity was considered by using a 6-order Prony series identified from high frequency data by Nicolle et al. (2005)

In the formula, *N* refers to the number of selected voxels for a finite element, and $\mathbf DiffT _i$ and $D_i$ are respectively the diffusion tensor and the normalized distance from the center of the voxel to the center of the finite element for each selected voxel (*i*).

**Fig. 6:**
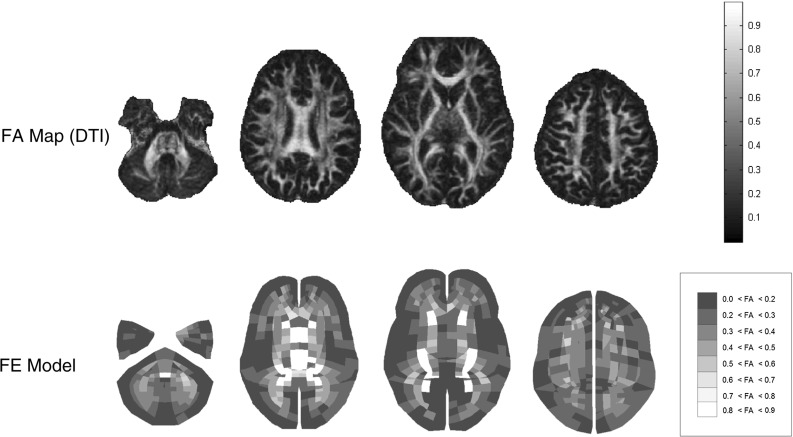
Separation between gray and white matter based on fractional anisotropy from DTI. The image refers to the baseline model by Giordano and Kleiven (2014). No elements are found in the most anisotropic group 0.9 < FA < 1.0. From the *left* to the *right*, axial cross sections of the FE model and FA map at *z* = −46.9, −11.2, −3.3 and 28.4 mm are illustrated. The *z*-axis represents the inferior–superior direction and coordinates are expressed relatively to the center of mass of the head

The white matter tract orientation was extracted as the principal eigenvector of $\mathbf DiffT _i$, and was specified in the model element-wise by a system of local coordinates using the ${}^*$ ELEMENT_ SOLID_ORTHO keyword in the Ls-Dyna input deck. In terms of model parts, the brain was divided into gray matter and eight groups of white matter based on FA values (Table 2; Fig. 6).

### Loading conditions

The kinematics from reconstructions of a sport accident that took place in the American National Football League (case NFL57) (Newman et al. 2000) were imposed on the FE models. This specific case was selected because of high rotational acceleration occurring in the transversal and coronal planes, which is typically associated with traumatic brain injury. The accident dynamic consisted in a football player striking another football player in the head. The striking player was not injured while the struck player was concussed.

**Fig. 7:**
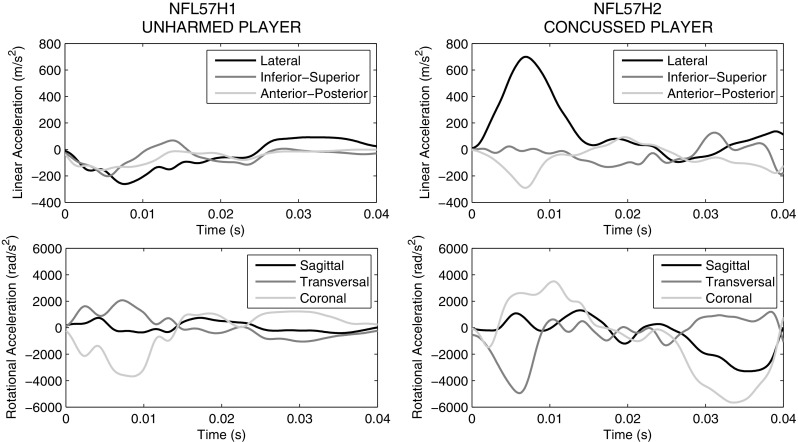
Head models loading conditions based on the reconstruction of a concussive case (case study NFL57) (Newman et al. 2000). Translational and angular accelerations are illustrated for both concussed and unharmed players

In all the simulations, the skull was a rigid body while translational and rotational accelerations (Fig. 7) were applied to the head center of gravity. The interface between the dura mater and the skull was modeled in LsDyna with tied-surface contacts. The meninges–brain interface was instead modeled with a sliding only contact that did not allow any separation in the radial direction but allowed transfer of tension and compression in the radial direction. This modeling choice wanted to represent the vacuum experienced by the cerebrospinal fluid when inertia forces created tension in brain region opposite to the impact location.

The GOH model was supplied to Ls-Dyna as a user subroutine. The custom executable was called in the input deck using a user-defined material with input parameters from Tables 1, 2 and 3. For the calculations, reduced integration and hourglass control were used for the brain while selectively reduced integration was used for the other components. The hourglass energies were always controlled to be lower than 10% of the peak internal energy for each part in the model.

### Strain-based injury criteria

Finite element head impact analyses were used in combination with axonal strain-based injury predictors to assess the risk of traumatic brain injury. In the current study, the peak values of the principal Green-St. Venant strain and axonal strain were considered independent of the time of occurrence. The peak value was selected because it represented the worst case scenario, namely the maximum deformation that occurred in the tissue and that was believed to cause the injury.

Specifically, the axonal strain expressed the deformation of the tissue in the direction of white matter tracts and was defined as

9 $$\begin{aligned} \begin{aligned}&\tilde{E}_{\alpha }=k(\tilde{I}_1-3)+(1-3k)(\tilde{I}_{4a}-1)\\&\tilde{I}_{4a}=\tilde{\mathbf{C }} : \mathbf n _{0a} \otimes \mathbf n _{0a} \end{aligned} \end{aligned}$$

where $\tilde{E}_{\alpha }$ represents the axonal strain, $\tilde{I}_1$ corresponds to the first invariant of the isochoric Cauchy–Green strain tensor, $\tilde{\mathbf{C }}$  is the isochoric Cauchy–Green strain tensor, $\mathbf n _{0a}$ the fiber unit vector in the undeformed configuration and *k* the dispersion parameter. This measure was directly calculated from the constitutive model for the brain tissue, thanks to the anisotropic formulation of the material.

**Table 4:** Mean, standard deviation and coefficient of variation of FA for the 15 ROIs analyzed in the study

ROI	FA Mean	FA SD	FA Cv (%)
GCC	0.5585	0.0406	7.2631
BCC	0.5532	0.0473	8.5534
SCC	0.6446	0.0518	8.0409
CSTR	0.4287	0.0502	11.7033
CSTL	0.4359	0.0481	11.0422
MLR	0.4997	0.0494	9.8977
MLL	0.4968	0.0511	10.2975
ACRR	0.4011	0.0286	7.1311
ACRL	0.4212	0.0279	6.6283
SCRR	0.4208	0.0315	7.4956
SCRL	0.4408	0.0379	8.5919
PCRR	0.4249	0.0324	7.6276
PCRL	0.4114	0.0351	8.5392
PTRR	0.5156	0.0362	7.0299
PTRL	0.5487	0.0411	7.4978

The mean was operated subject-wise and ROI-wise (see Fig. 2)

**Fig. 8:**
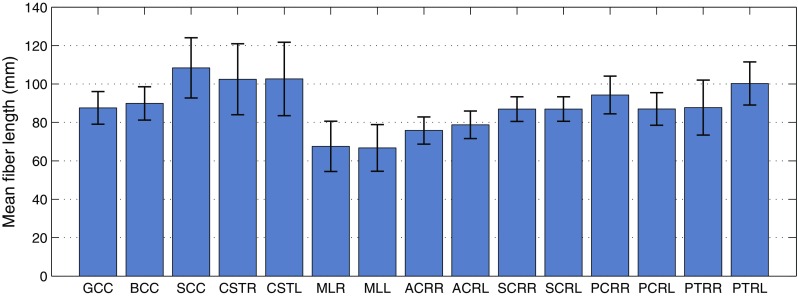
Mean length of the fibers corresponding to the 15 selected ROIs for TBI analysis. Error bars represent the inter-subject variability over 485 subjects (SD)

## Result

### Statistical analysis of white matter tracts

The normalization procedure of the 485 diffusion images showed a mean registration error ± inter-subject variability of $2.15 \pm 0.18\%$. In addition, above $57\%$ of the voxels that presented different values between the registered and the fixed images were false positives, meaning that the subject’s registered images were slightly bigger than the MNI152 template. Table 4 reports the inter-subject variability of FA for the commissural fibers of the corpus callosum, cortico-spinal tract, medial lemniscus, corona radiata, and posterior thalamic radiation. The mean values ranged from $0.4011 \pm 0.0286$ in the anterior right corona radiata to $0.6446 \pm 0.0518$ in the splenium of the corpus callosum. The thalamic radiation and the corpus callosum showed relatively high anisotropy values compared with those of the corona radiata. The biological variability was found to be elevated with a mean coefficient of variation across the ROIs of 8.48%. In the analysis of interhemispheric differences, significant left–right asymmetry was not found. Minor differences in mean values were present in several regions, most significantly in the posterior thalamic radiation. However, in general, the differences in regional mean values were moderate and within 1 standard deviation (SD).

Figure 8 reports the mean length of the fibers seeded in the 15 regions of interest for TBI. The mean length values ranged from $66.72 \pm 12.17$ mm (left medial lemniscus) to $108.4 \pm 15.69$ mm (splenium of the corpus callosum). The biological variability was found to be high with a mean coefficient of variation across the ROIs of 12.6%.

**Fig. 9:**
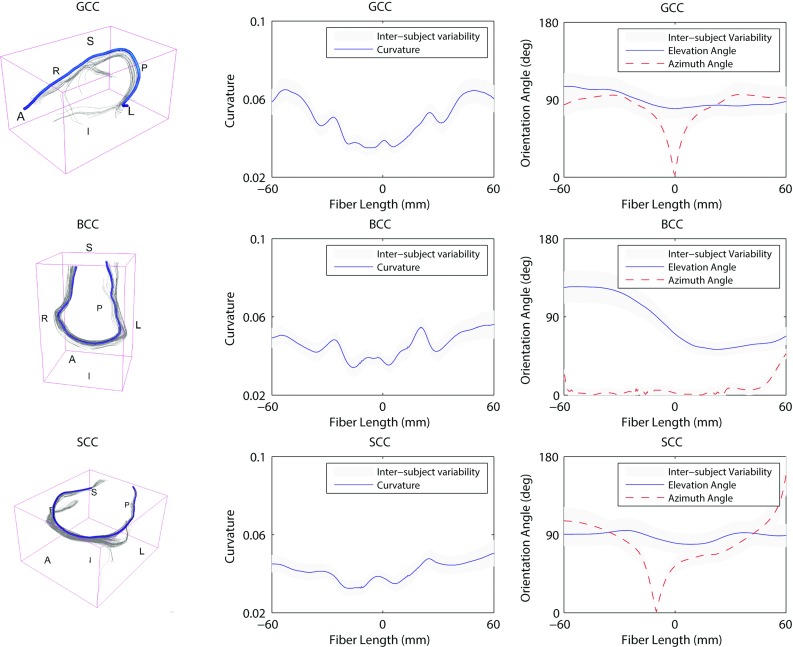
Inter-subject variability of the 3D orientation of the callosal white matter tracts. *On the left* visualization in the 3D space of the reference fiber (*A* anterior, *P* posterior, *L* left, *R* right). *In the middle* curvature along the reference fiber. *On the right* evolution of the elevation angle and azimuth angle (refer to Fig. 4). The inter-subject variability is represented as a *gray band* indicating ± 1 SD. The origin of the plot corresponds to the center of the ROI. Negative length values are related to fibers in the left part of the ROI

**Fig. 10:**
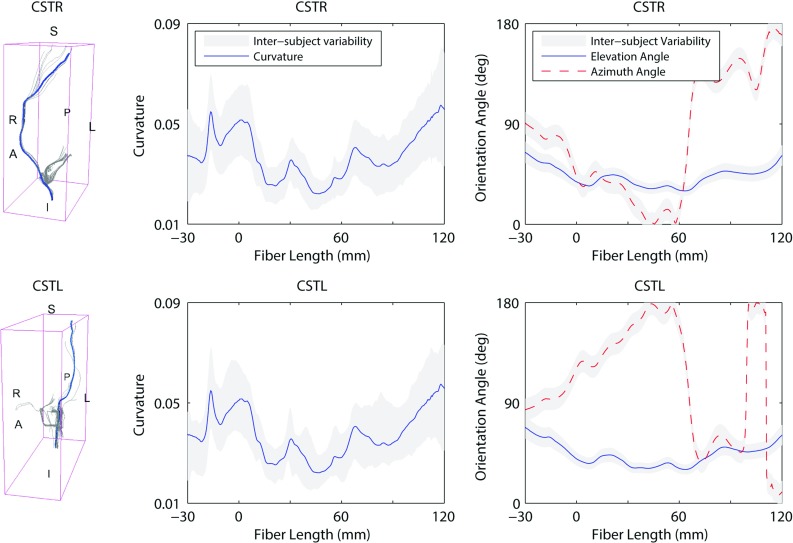
Inter-subject variability of the 3D orientation of the cortico-spinal tracts. *On the left* visualization in the 3D space of the reference fiber (*A* anterior, *P* posterior, *L* left, *R* right). *In the middle* curvature along the reference fiber. *On the right* evolution of the elevation angle and azimuth angle (refer to Fig. 4). The inter-subject variability is represented as a *gray band* indicating ± 1 SD. The origin of the plot corresponds to the center of the ROI. Negative length values are related to fibers in the inferior part of the ROI

Figures 9 and 10 illustrate the white matter tract geometrical characteristics along the reference fiber for the corpus callosum and the cortico-spinal tracts respectively. The detailed results for all the 15 ROIs are reported in “Appendix 2.” On the left of Figs. 9 and 10, the tractography model allows the visualization of the fibers in 3D space. In the middle, the average curvature along the reference fiber is reported. On the right, the evolution of the elevation angle ($\varTheta$) and azimuth angle ($\varPhi$) (Fig. 4) is depicted to show the distribution of the reference fiber along the parametrized arc. The inter-subject variability is represented as a *gray band* indicating ± 1 SD. For the corpus callosum (Fig. 9) the fibers were analyzed laterally, from left to right. For the cortico-spinal bundles (Fig. 10) the analysis was performed vertically, from inferior to superior. The origin of the plot corresponds to the center of the ROI.

As it can be seen from Figs. 9 and 10, the inter-subject variability for curvature was found to be high. The mean curvature for the callosal fibers of the genu, body and splenium was $0.052 \pm 0.0058$, $0.047 \pm 0.0054$ and $0.043 \pm 0.0047$, respectively. The coefficient of variation across the ROIs was 11.16%. The cortico-spinal bundles showed a mean curvature of $0.039 \pm 0.014$ for the left and $0.038 \pm 0.013$ for the right bundles. The coefficient of variation across the ROIs was 36.23%.

The evolution of the elevation ($\varTheta$) and azimuth ($\varPhi$) angles along the reference fiber (Fig. 4) also showed significant inter-subject variability. On average, the elevation angle for the callosal fibers of the genu, body and splenium was $90.21^{\circ } \pm 8.85^{\circ }$, $86.35^{\circ } \pm 28.54^{\circ }$ and $88.46^{\circ } \pm 4.62^{\circ }$, respectively. $\varTheta$ had a coefficient of variation across the ROIs of 20.13%. The mean azimuth angle was $82.21^{\circ } \pm 8.85^{\circ }$ for the genu, $11.54^{\circ } \pm 6.17^{\circ }$ for the body and $88.46^{\circ } \pm 4.62^{\circ }$ for the splenium of the corpus callosum. The coefficient of variation for $\varPhi$ across the ROIs was 45.38%. The cortico-spinal bundles showed a mean elevation angle of $42.79^{\circ } \pm 8.38^{\circ }$ for the right and $43.56 \pm 9.67$ for the left bundles. $\varTheta$ differed across the ROIs with a coefficient of variation of 20.88%. On average, the azimuth angle of the right and left bundles was $77.47^{\circ } \pm 14.18^{\circ }$ and $110.82^{\circ } \pm 41.40^{\circ }$, respectively. The coefficient of variation across the ROIs was 27.83%.

### Subject-specific FE models

Subject-specific anisotropic FE models were generated by mapping the diffusion information of a single subject within the baseline FE model. According to Giordano et al. (2014), the brain was divided in gray and white matter and, in turn, white matter was divided in eight groups based on the alignment of the neuronal fibers (Table 2). According to Eq. 8, diffusion information from medical images was averaged to extract the mean anisotropy information for each finite element. Table 5 illustrates how the inter-subject biological variability of FA from medical images translated into variability in the FE models after the weighted averaging procedure. The finite elements corresponding to the commissural fibers of the corpus callosum, cortico-spinal tract, medial lemniscus, corona radiata, and posterior thalamic radiation were identified based on spatial correspondance. The mean FA values ranged from $0.3648 \pm 0.0272$ in the posterior right corona radiata to $0.6084 \pm 0.0515$ in the splenium of the corpus callosum. Although some smoothing effects were visible due to the procedure (typically the FA mean values were lower in the model than in the medical images), the biological variability was found to be representative of the medical images with a mean coefficient of variation across the ROIs of 8.27%.

**Fig. 11:**
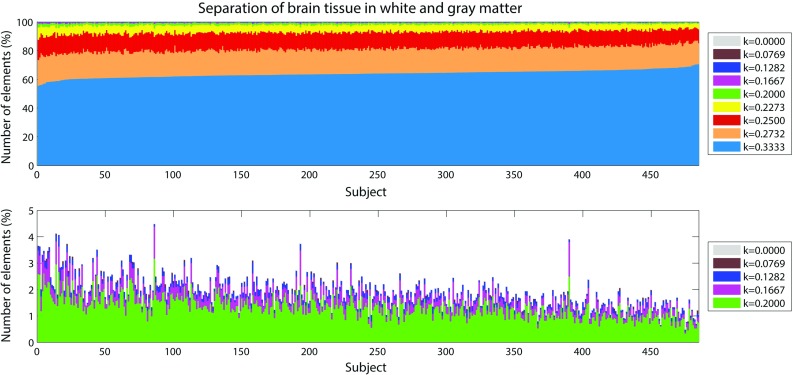
Proportion of finite elements belonging to a certain group of gray/white matter in the brain for the 485 subjects. *On the top* results for the nine groups of brain tissue. *k* $=$ 0.3333 corresponds to gray matter. *At the bottom* zoom of the results for the most anisotropic groups

Figure 11 illustrates the proportion of finite elements belonging to a certain anisotropy group in the brain for the 485 analyzed subjects. As it can be seen from Fig. 11, the biological variability of white matter tracts affected the determination of the mechanical fiber alignment parameter *k* and the separation of gray and white matter in the FE model. The average number of elements belonging to gray matter ($k=0.3333$) ranged from 2291 (55.55%) to 2918 (70.75%) with a coefficient of variation of 3.8%.

**Fig. 12:**
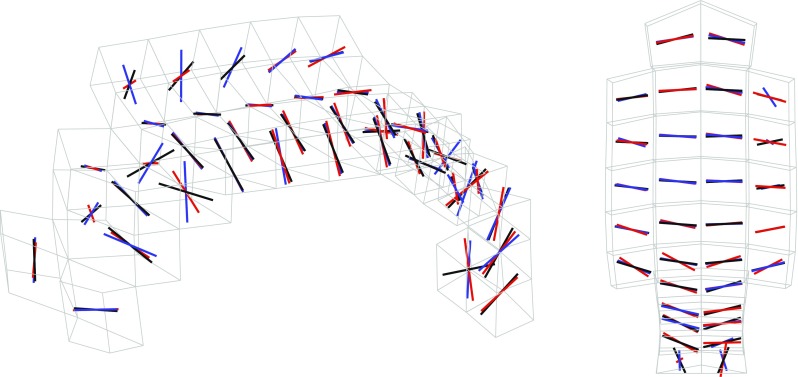
Orientation of white matter tracts in the corpus callosum of three subjects (*black*, *blue* and *red*) randomly chosen in the pool of 485

The biological variability of white matter tracts also affected the orientation of the fiber reinforcements in the FE model. As a mean of example, Fig. 12 illustrates the orientation of white matter tracts in the corpus callosum of three subjects randomly chosen in the pool of 485. Similar to Eq. 4, to measure the statistical variability of the orientations over the 485 analyzed subjects, the fiber principal orientation vector **P** was characterized in terms of elevation ($\varTheta$) and azimuth ($\varPhi$) angles:

10 $$\begin{aligned} \mathbf P (\varTheta , \varPhi )={\sin }(\varTheta ){\cos }(\varPhi )\mathbf e _1 + {\sin }(\varTheta ){\sin }(\varPhi )\mathbf e _2 + {\cos }(\varTheta )\mathbf e _3 \end{aligned}$$

The biological variability was found to be considerable with the mean coefficient of variation across the white matter equal to 39.42% for $\varTheta$, elevation angle, and 29.31% for $\varPhi$, azimuth angle.

**Table 5:** Mean, standard deviation and coefficient of variation of FA in the finite elements corresponding to the 15 ROIs analyzed in the study

Finite elements	FA mean	FA SD	FA Cv (%)
GCC	0.4799	0.0405	8.4370
BCC	0.5178	0.0460	8.8876
SCC	0.6084	0.0515	8.4624
CSTR	0.3788	0.0395	10.4351
CSTL	0.3818	0.0390	10.2249
MLR	0.4369	0.0494	11.3077
MLL	0.4435	0.0439	9.9014
ACRR	0.3769	0.0280	7.4391
ACRL	0.3888	0.0248	6.3820
SCRR	0.4082	0.0273	6.6950
SCRL	0.3958	0.0237	5.9841
PCRR	0.3648	0.0272	7.4566
PCRL	0.3654	0.0286	7.8255
PTRR	0.4728	0.0349	7.3801
PTRL	0.5002	0.0359	7.1818

The mean was operated subject-wise and ROI-wise (see Fig. 2)

### Traumatic brain injury prediction

**Fig. 13:**
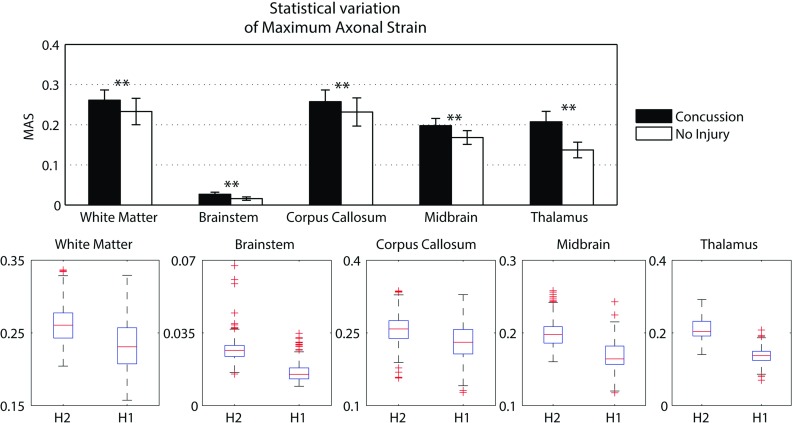
A summary of the mean values and standard deviations observed in the simulations for axonal strain. The values are shown in *black* for concussed ($$n=485$$) and white for non-injured ($$n=485$$) players for various anatomical regions of the brain. Statistical differences in *t* tests are reported as $$**$$ when $$p<$$0.05. The *boxplots* show the median, minimum, and maximum value for axonal strain. Outliers are plotted as crosses outside the *box*

**Fig. 14:**
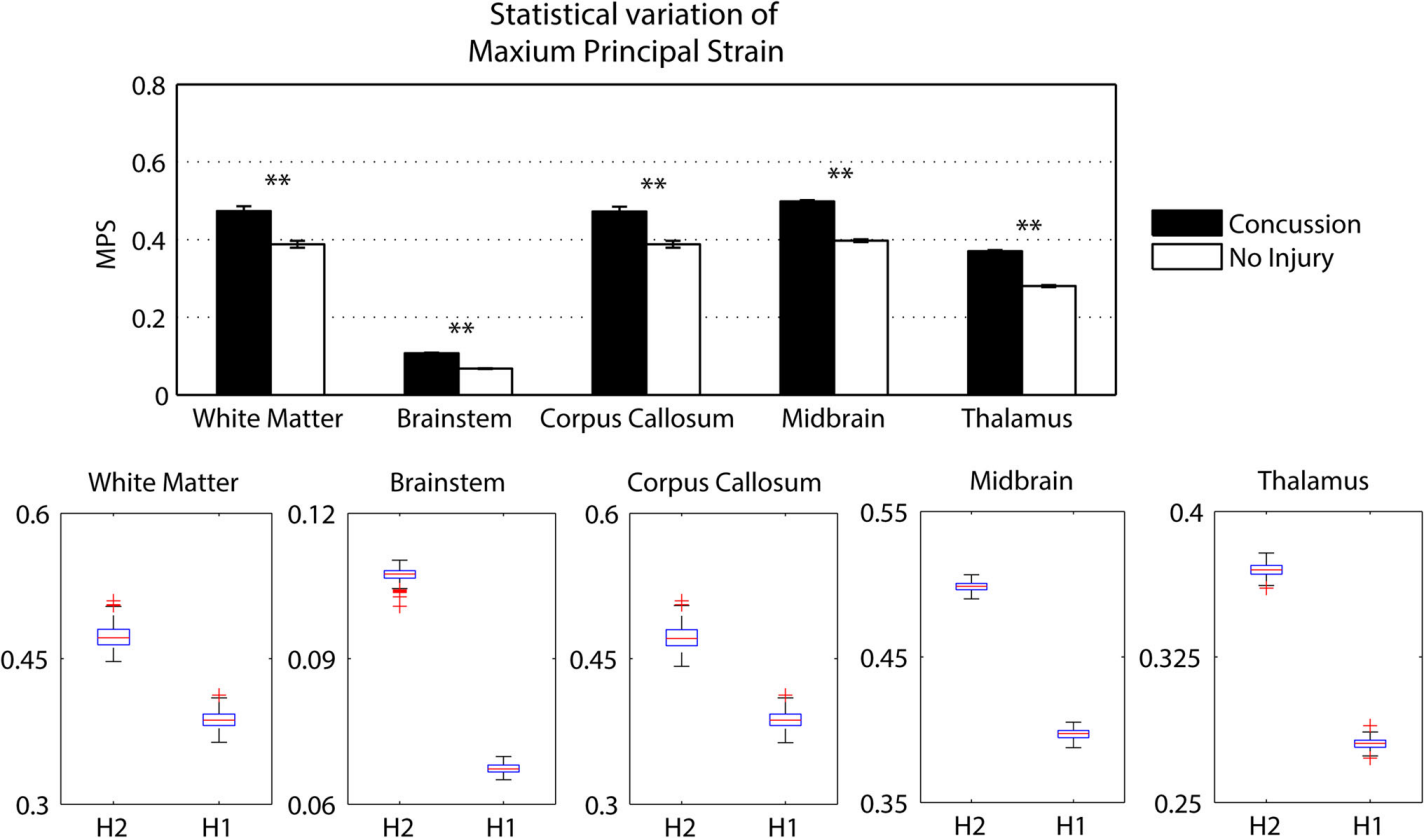
A summary of the mean values and standard deviations observed in the simulations for principal strain. The values are shown in *black* for concussed ($$n=485$$) and *white* for non-injured ($$n=485$$) players for various anatomical regions of the brain. Statistical differences in *t* tests are reported as $$**$$ when $$p<$$0.05. The *boxplots* show the median, minimum, and maximum value for principal strain. Outliers are plotted as crosses outside the *box*

A summary of the model prediction of maximum axonal strain (MAS) for different areas of the brain is reported in Fig. 13. For comparison, a summary of the model prediction of maximum principal strain (MPS) is reported in Fig. 14. The mean values and standard deviations observed in the simulations are illustrated for concussed and uninjured players in the whole white matter, brainstem, midbrain, corpus callosum, and thalamus. For the concussed player, the mean values for MAS ranged from $0.027 \pm 0.0051$ in the brainstem to $0.261 \pm 0.0253$ in the whole white matter. The mean values for MPS ranged from $0.107 \pm 0.0013$ in the brainstem to $0.498 \pm 0.0034$ in the midbrain. For the unharmed player, the mean values for MAS ranged from $0.016 \pm 0.0042$ in the brainstem to $0.233 \pm 0.033$ in the whole white matter. The mean values for MPS ranged from $0.067 \pm 0.001$ in the brainstem to $0.397 \pm 0.003$ in the midbrain. Overall, the variability of the model predictions was found to be high with a mean coefficient of variation across the whole white matter of 11.91% for MAS and 2.33% for MPS.

**Fig. 15:**
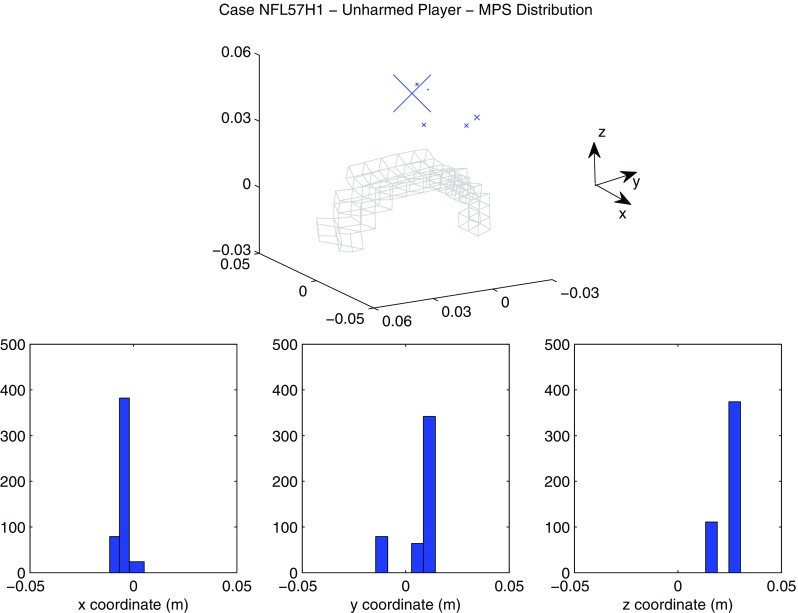
Location of maximum principal strain for the unharmed player (Case NFL57H1). *On the top* *scatter plot* representing the nodes where the maximum principal strain was observed. The size of the markers (*blue*) is proportional to the frequency of occurence of a certain location. For spatial reference, the mesh of the corpus callosum is plotted in *gray*. *On the bottom* distribution of the coordinates of the points where maximum principal strain was found. *X*-axis, *Y*-axis, and *Z*-axis correspond to the lateral, anterior–posterior and inferior–superior directions, respectively

**Fig. 16:**
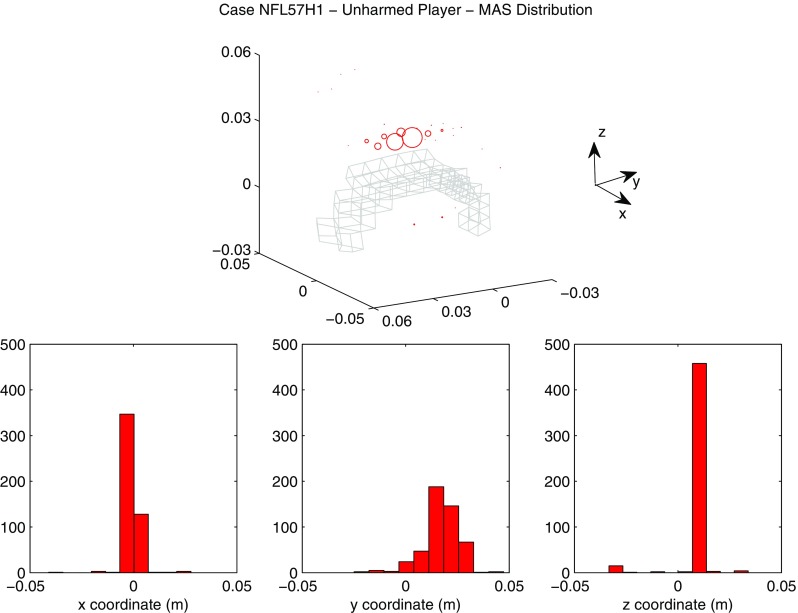
Location of maximum axonal strain for the unharmed player (Case NFL57H1). *On the top*: *scatter plot* representing the nodes where the maximum axonal strain was observed. The diameter of the markers (*red*) is proportional to the frequency of occurence of a certain location. For spatial reference, the mesh of the corpus callosum is plotted in *gray*. *On the bottom* distribution of the coordinates of the points where maximum axonal strain was found. *X*-axis, *Y*-axis and *Z*-axis correspond to the lateral, anterior–posterior, and inferior–superior directions, respectively

**Fig. 17:**
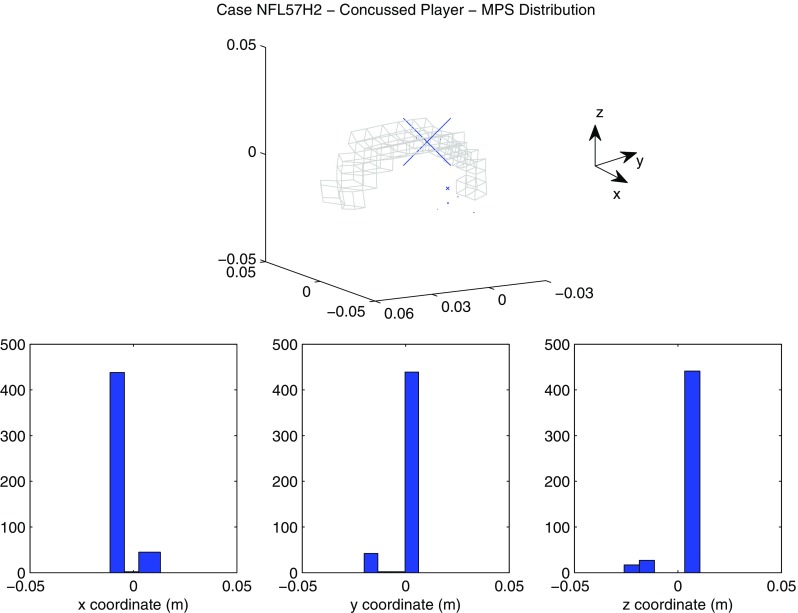
Location of maximum principal strain for the concussed player (Case NFL57H2). *On the top* *scatter plot* representing the nodes where the maximum principal strain was observed. The size of the markers (*blue*) is proportional to the frequency of occurence of a certain location. For spatial reference, the mesh of the corpus callosum is plotted in *gray*. *On the bottom*: distribution of the coordinates of the points where maximum axonal strain was found. *X*-axis, *Y*-axis, and *Z*-axis correspond to the lateral, anterior–posterior, and inferior–superior directions respectively

**Fig. 18:**
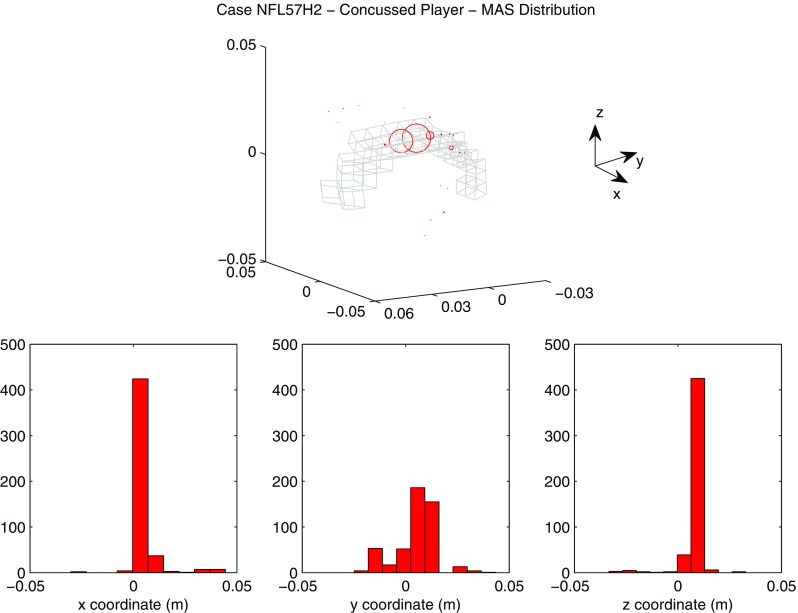
Location of maximum axonal strain for the concussed player (Case NFL57H2). *On the top* *scatter plot* representing the nodes where the maximum axonal strain was observed. The diameter of the markers (*red*) is proportional to the frequency of occurence of a certain location. For spatial reference, the mesh of the corpus callosum is plotted in *gray*. *On the bottom* distribution of the coordinates of the points where maximum axonal strain was found. *X*-axis, *Y*-axis, and *Z*-axis correspond to the lateral, anterior–posterior and inferior–superior directions, respectively

The *t* test for equality of means was used for a statistical comparison between the concussed (Figs. 13, 14 black) and the unharmed (Figs. 13, 14 white) populations. A statistical difference (*t* test, $p<$ 0.05) was found in all areas of the brain meaning that, although the prediction of MAS and MPS were affected by biological variability, the mean values of the two populations (unharmed/concussed) were still different within the 5% confidence interval. The FE models predicted a typical strain pattern in the brain, independent of the subject specificity of the model. For the unharmed player, areas of large principal strain were observed in the temporal lobe and superior parts of the cortex (Fig. 15). The maximum axonal strain was mostly located in the cingulate gyrus (Fig. 16). For the concussed player, areas of large principal strain were observed in the splenium of the corpus callosum as well as in the temporal and occipital lobe close to the right lateral ventricle (Fig. 17). A similar strain pattern was seen for the axonal strain whose peaks were mostly located at the edge between the body of the corpus callosum and the lateral ventricles (Fig. 18). Figures 15, 16, 17 and 18 illustrate the localization of MPS and MAS for the concussed and unharmed player, respectively, over the 485 studied subjects. As indicated by the figures, despite the biological variability, the measures of MPS and MAS were repeatable. Disregarding the outliers, the MAS was located in a 2 cm^3^ volume: laterally (*x*-axis) and vertically (*z*-axis) coordinates varied within 1 cm while horizontally (*y*-axis) the variation was slightly larger, mostly within 2 cm (Figs. 16,18).

## Discussion

### Biological variability of white matter tracts

At present, only a few reports of biological variability data for regional FA are available (Lee et al. 2009; Oishi et al. 2008; Yendiki et al. 2011; Veenith et al. 2013). The cumulative data derived from this kind of studies forms the basis for the use of DTI measurements in clinical patients, where, a sound definition of healthy state is necessary prior to the diagnosis/classification of a disease. The data derived from this study therefore contribute to define FA values in a normal population. This diffusion information can be used in future studies to observe alterations in the values of diffusion parameters related to white matter pathologies.

When compared to reports of measurements made by Oishi et al. (2008) and Yendiki et al. (2011), the estimates in the corpus callosum, brainstem, thalamus, and corona radiata showed agreement within a standard deviation. However, when compared to reports of measurements made by Lee et al. (2009) and Veenith et al. (2013), the results obtained in the current study differed to a greater extent from the literature (mostly within 2–3 SD). The dissimilarities were probably due to differences in the choice of the ROI’s location as well as in the choice of the reference space used for image normalization. As a matter of fact, in Oishi’s study (Oishi et al. 2008) the ICBM-DTI-81 atlas in MNI space was used as reference, as it was done in the present study. In Yendiki’s study (Yendiki et al. 2011), ROIs were manually labeled but the reference frame for image normalization was, once again, the MNI-152 atlas. In Lee’s and Veenith’s studies (Lee et al. 2009; Veenith et al. 2013) other spatial references were adopted. It is concluded that using a common reference space for image normalization produced diffusion parameters mean values in agreement within a standard deviation. If significant differences in FA values are found between two studies, it could be due to consistent anatomical difference between the two spatial references. Moreover, for areas of the brain located close to the ventricles, differences could be due to cerebrospinal fluid (CSF) contamination, i.e., to the inclusion of voxels representing CSF in the average values extracted, due to incorrect normalization.

In order to study the microstructural organization of the brain tissue in a healthy population, tractographic data were analyzed in terms of mean length, orientation and curvature. The mean length of the fibers of the corpus callosum was $87.57 \pm 8.53$ mm for the genu, $89.91 \pm 8.68$ mm for the body and $108.4 \pm 15.69$ mm for the splenium. These values were comparable within 2 SD to the results by Caminiti et al. (2013), where the length of the callosal fibers (2$\times$ length from the cerebral cortex to the fiber midline) was reported to be 110.40, 114.74, and 142.44 mm for the genu, the body and the splenium respectively. In Caminiti’s study, a slightly overestimation of the length occurred since the distance between the end of the fiber and the layer III of the cortex was included in the calculations.

Overall fibers resulted longer in the posterior part of the structures, in contrast with the anterior: it can be noted in Fig. 8 for the corpus callosum and corona radiata. Moreover, the central part of the brain structures presented lower variability than the peripheral one. The fiber in the brainstem presented the highest variability. Registration errors in image normalization could partly explain the higher variability, being the brainstem located at the edge of the registered images.

The evolution of the elevation and azimuth angles for the callosal and cortico-spinal fibers indicated fiber shapes in agreement with the expected 3D distribution known from histology. The fibers seeded from the genu of the corpus callosum were characterized by an elevation angle of circa $90^{\circ }$ and an azimuth angle varying in the range $90^{\circ }$–$0^{\circ }$–$90^{\circ }$ (Fig. 9) . This indicated *u*-shaped tracts with most fibers lying in the transverse plane. The tracts seeded from the body of the corpus callosum (Fig. 9) presented small azimuth angles. The elevation angle varied between $120^{\circ }$ and $60^{\circ }$. This represented *u*-shaped white matter tracts with disposition in the coronal plane. The splenium had a shape similar to the genu with some tracts lying on oblique surfaces between the transversal and coronal planes. Both the left and right cortico-spinal tracts were characterized by elevation angles of circa $45^{\circ }$ (Fig. 10) indicating disposition in the coronal plane with a moderate inclination with respect to the vertical. Furthermore the trend of the azimuth angles represented first a distribution of the fibers toward the anterior–posterior direction followed by a progressive separation of the bundles toward the left and right directions respectively.

The curvature of the white matter tracts presented significant biological variability. Generally, the variability increased with the distance from the seed point, where the track estimation was started. This could indicate that the effects of the biological variation may have summed up with propagation errors of the tracking algorithm. In particular, from Figs. 9 and 10 it can be noticed that the callosal fibers of the genu showed the highest curvature with respect to the other tracts of the corpus callosum (marked *u* shape). Meanwhile, the tracts from the body presented the highest curvature variability. Moreover the callosal fibers showed significant lower mean curvature variability than the average among all the regions (0.016). This result is in agreement with the highest percentage of overlap of the callosal fibers reported in Bürgel and Amunts (2006) and Schotten et al. (2011). The cortico-spinal bundles did not present significant differences in the curvature between the right and left tracts. It is worth noting that the inter-subject variability was significant within the seed point (i.e., the brainstem) and at 40 mm after the origin, around the midbrain.

The 3D orientation of the white matter bundles also showed considerable biological variability. The coefficient of variability of the orientation angles varied between 20 and 45%. The standard deviation typically increased with the distance from the seed point, where the track estimation was started. A major limitation of the statistical analysis of white matter tract was the error introduced by the reference fiber-based correspondence criterion used for clustering of tracts. The comparison of the fibers was indeed done by aligning all the fibers in a ROI with the reference fiber, seeded from the center of the ROI. The procedure assumed similarity of fiber shapes in a ROI. Indeed, the labels of the JHU atlas were defined on the base of the principal diffusion eigenvector (Jenkinson 2015); therefore, white matter fibers in a region should have had similar spatial distribution. However, it was noticed that, for bigger ROIs, few tracts with different three-dimensional shape were forced to correspond independently of their shape. In future studies, more precise result may be obtained by including clustering of different families of fibers before alignment to the reference.

### Traumatic brain injury prediction

Computational models incorporating anisotropic features of brain tissue have become a valuable tool for studying the development of TBI. The tissue deformation in the direction of white matter tracts (axonal strain) was repeatedly shown to be an appropriate mechanical parameter to predict injury (Sahoo et al. 2015; Giordano and Kleiven 2014). In particular, when anisotropic metrics are promoted as injury predictors, the uncertainty of the predictions due to biological variation should be carefully investigated. As a matter of fact, a variation in white matter tract orientation would affect the orientation of the fiber reinforcement in the anisotropic FE model, the value of diffusion parameters based on which mechanical parameters are determine and, ultimately, the model responses and injury prediction results. This means that, on top of the modeling uncertainties due to the approximation of the geometry and material properties, the FE analysis will be affected by an extra source of uncertainty. This is important to consider when assessing the reliability of axonal strain to predict TBI in a population.

The present study investigated the sensitivity of axonal strain to biological in DTI data of a population of 485 healthy subjects. It was observed that the biological variability affected the mechanical response of the white matter subject-specific FE models. According to Giordano et al. (2014), the separation of brain tissue into gray and white matter was based on FA values from diffusion tensor images. As expected, the biological variability of FA (Table 4) affected in cascade the coupling procedure generating subject-specific models with a different percentage in volume of white matter and different alignment of the fibers (Figs. 11, 12). The volume percentage of gray matter in the model showed a coefficient of variation equal to 3.8% with gray matter variating from 55.55% of the total brain volume to the 70.75%. Also, the orientation of the fiber reinforcements in the FE model had a coefficient of variation of 39.41% for the elevation angle and 29.31% for the azimuth angle (Eq. 9). Finally, the coefficient of variation of 9.86% for mean FA in medical images turned into a coefficient of variation of 8.26% for the correspondent finite elements in the models (Tables 4 vs. 5). This variability reduction was likely due to the usage of an averaging procedure to map the diffusion information to the model (Giordano et al. 2014). Finite elements with a mean length of 5.8 mm were indeed associated to DTI voxels with a length of 1 mm. A consequence of applying such a voxel mean calculation was a smoothing of the diffusion parameters between elements. Given the coarse mesh of KTH model, this procedure was necessary and was the biggest limitation of this study. Nevertheless, although some smoothing effects were visible (typically the FA mean values were lower in the model than in the medical images), the subject-specific models still differed importantly from one another and the model inter-subject variability was found to be representative of the medical images (similar coefficients of variation).

The magnitude of principal and axonal strain in the brain was larger for the concussed player than for the unharmed player. This confirmed a positive correlation between the risk of injury and the magnitude of deformation of the tissue. For the unharmed player, areas of large principal strain were observed in the superior parts of the cortex (Fig. 15). The maximum axonal strain was mostly located in the cingulus gyrus (Fig. 16). In the concussive case, areas of large principal strain were observed in the posterior part of the corpus callosum close to the right lateral ventricle (Fig. 17). A similar strain patter was seen for the axonal strain (Fig. 18). These findings were in agreement with the centripetal theory of concussion (Gennarelli 2015) stating that, during a concussion, the disruption of the brain always begin at the surfaces and extend inwards to affect the diencephalic–mesencephalic core at the most severe levels of trauma.

As expected, the biological variability of white matter tracts affected the model predictions. The mean coefficient of variation across the whole white matter was 11.91% for MAS and 2.33 % for MPS (Figs. 15, 16, 17, 18). Being an orientation-dependent parameter, MAS was typically more sensitive than MPS to variation of fiber alignment or orientation. As a matter of fact, the variation reported in Figs. 15 and 17 was merely a result of the constitutive anisotropy while the variation reported in Figs. 16 and 18 was the result of both the constitutive anisotropy and the projection of the strain onto different fiber directions. To test the capability of principal and axonal strain to discern between non-injurious and concussed populations, the *t* test for equality of means was performed. A statistical difference (*t* test, $p<$0.05) was found all areas of the brain meaning that, although the prediction of MAS and MPS were affected by biological variability, the mean values of the two populations (unharmed/ concussed) were still different within the 5% confidence interval. Interestingly, despite biological variation, the localization of the maximum axonal strain was consistent for most of the subjects (Figs. 16, 17, 18). Disregarding some outliers, perfectly normal in a healthy population, the MAS was typically located in a 2 cm^3^ volume with most of the variation occurring in the anterior–posterior direction.

Considered all the sources of uncertainty of such complex FE models, it was concluded that axonal strain is an appropriate mechanical parameter to predict traumatic brain injury. MAS indeed distinguishes between injured and healthy populations and the variability of 11.91% in the numerical solution is comparable to the uncertainties in the calculations due to head geometry approximations (Kleiven and Holst 2002; Ho and Kleiven 2009) or modeling choices in material properties (Ji et al. 2014).

## Conclusion

In the present study, diffusion parameter values for a healthy state of the brain tissue were defined by the analysis of DTIs of 485 subjects. The normalization of the images to the MNI152 template and the adoption of the JHU labeled atlas enabled the comparison of the results with other studies. The study showed the importance of adopting a common spatial refence for the tissue analysis. An accurate alignment between the ROIs and the white matter structures is mandatory, especially for regions nearby the ventricles.

The main objective of the study was to evaluate the sensitivity of axonal strain to biological variability in DTI data of a population of 485 healthy subjects. It was found that the biological variability of white matter tracts affected the orientation of the fiber reinforcement in the anisotropic FE models, the value of diffusion parameters based on which mechanical parameters were determined and, ultimately, the injury prediction results. Despite a quite significant biological variability, for a sport concussion between two players (Case NFL57, Newman et al. 2000), MAS was capable to discern between non-injurious and concussed populations in several areas of the brain. The strain patterns predicted by the FE models were consistent and the maximum deformation was typically located in a 2 cm^3^ volume of the brain. It is concluded that axonal strain is an appropriate mechanical parameter to predict traumatic brain injury.

Nevertheless, the importance of subject-specific geometry and material properties, including the subject-specific white matter tract distribution, cannot be neglected. The subject-specific FE models generated in this study differed importantly from one another in terms of white matter volume and fiber reinforcement orientation. Although the same kinematics was applied to all the 485 models, the strain predictions varied with coefficients of variation of 11.91% for MAS and 2.33% for MPS. These variations underlines the incapability of an average FE model to represent the whole population and the necessity to develop families of models to cover specific, maybe vulnerable, populations. This is especially true for anisotropic models where the biological variation affects the orientation of the preferential directions and, in turn, the mechanical response of the model.

## Figures and Tables

**Fig. 19:**
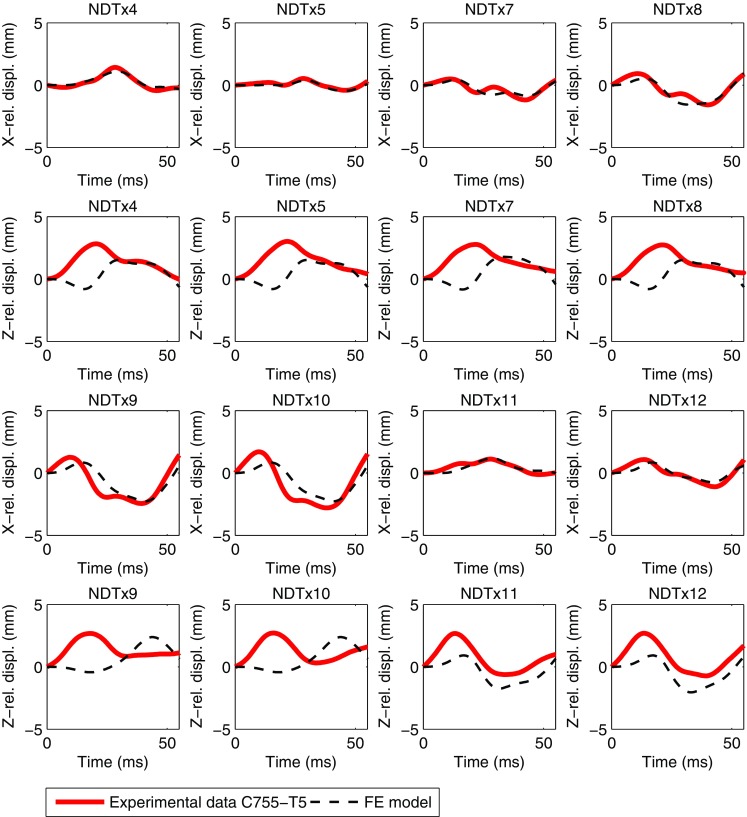
Simulation of relative skull-brain motion for the occipital impact C755-T5. For marker locations and coordinate system directions, see Hardy et al. (2001). Experimental data (*full lines*) are plotted together with the GOH anisotropic model responses (*dashed lines*)

**Fig. 20:**
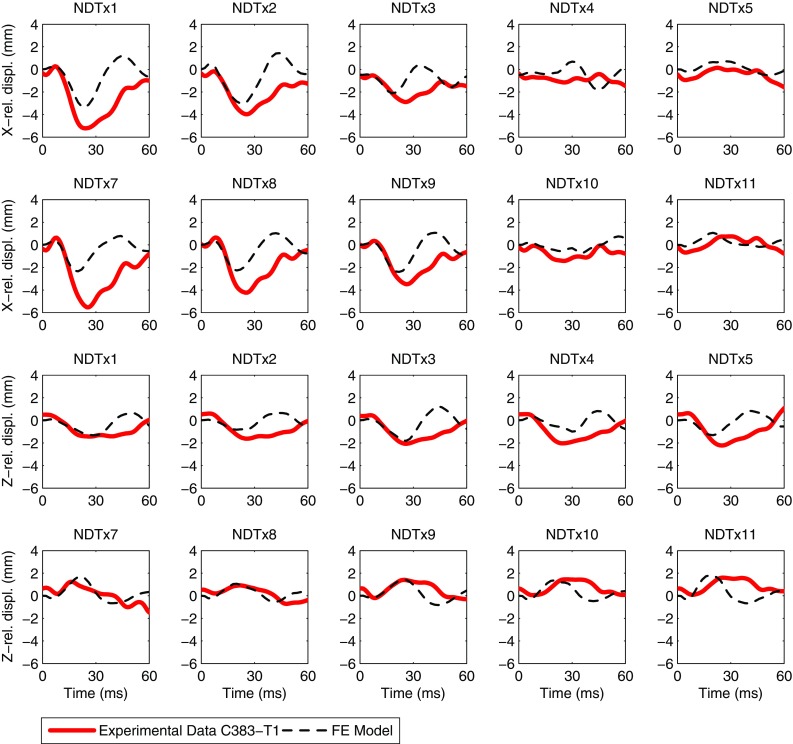
Simulation of relative skull-brain motion for the frontal impact C383-T1. For marker locations and coordinate system directions, see Hardy et al. (2001). Experimental data (*full lines*) are plotted together with the GOH anisotropic model responses (*dashed lines*)

**Fig. 21:**
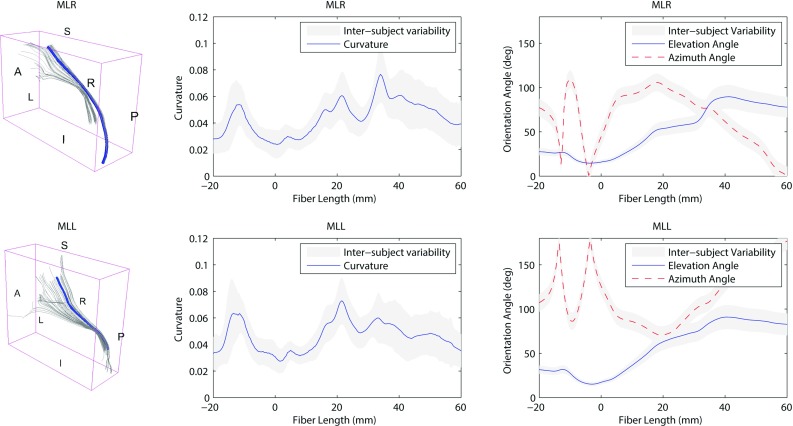
Inter-subject variability of the 3D orientation of the medial lemniscus (ML). *On the left* evolution of the components of the average tangent unit vector along the reference fiber. *On the right* average curvature along the reference fiber. The origin of the plot corresponds to the center of ROI

**Fig. 22:**
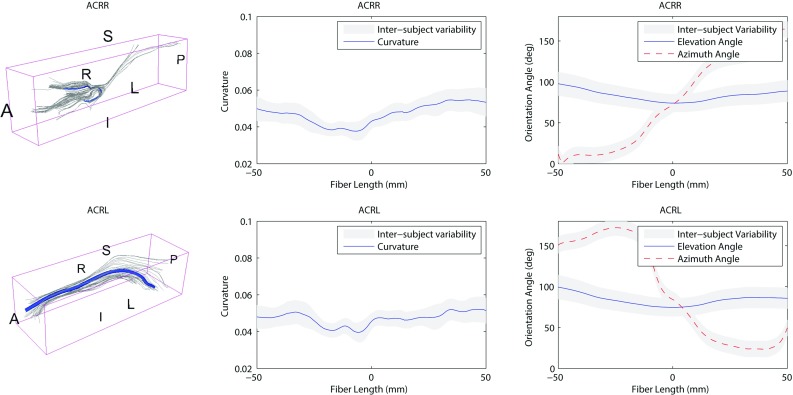
Inter-subject variability of the 3D orientation of the anterior corona radiata (ACR). *On the left* evolution of the components of the average tangent unit vector along the reference fiber. *On the right* average curvature along the reference fiber. The origin of the plot corresponds to the center of ROI

**Fig. 23:**
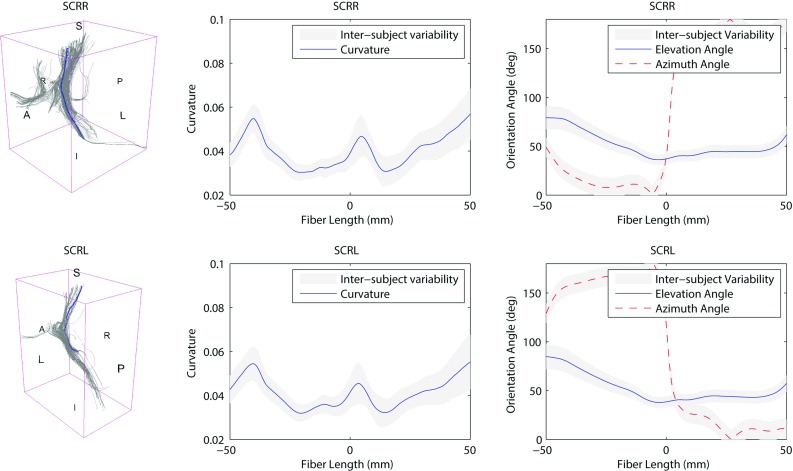
Inter-subject variability of the 3D orientation of the superior corona radiata (SCR). *On the left* evolution of the components of the average tangent unit vector along the reference fiber. *On the right* average curvature along the reference fiber. The origin of the plot corresponds to the center of ROI

**Fig. 24:**
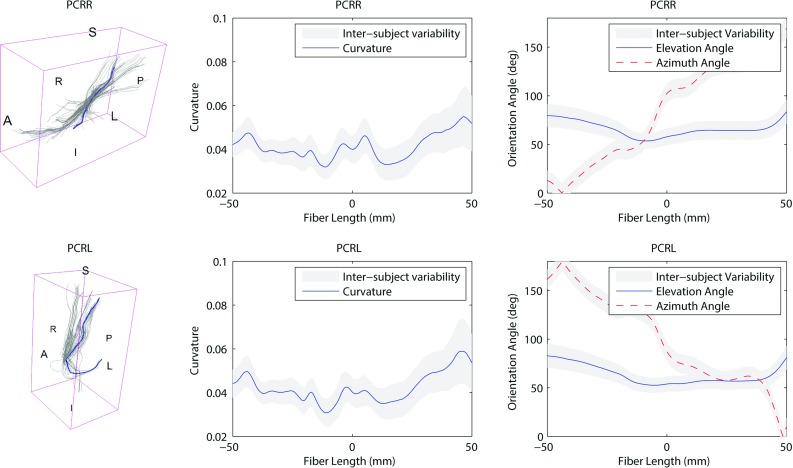
Inter-subject variability of the 3D orientation of the posterior corona radiata (PCR). *On the left* evolution of the components of the average tangent unit vector along the reference fiber. *On the right* average curvature along the reference fiber. The origin of the plot corresponds to the center of ROI

**Fig. 25:**
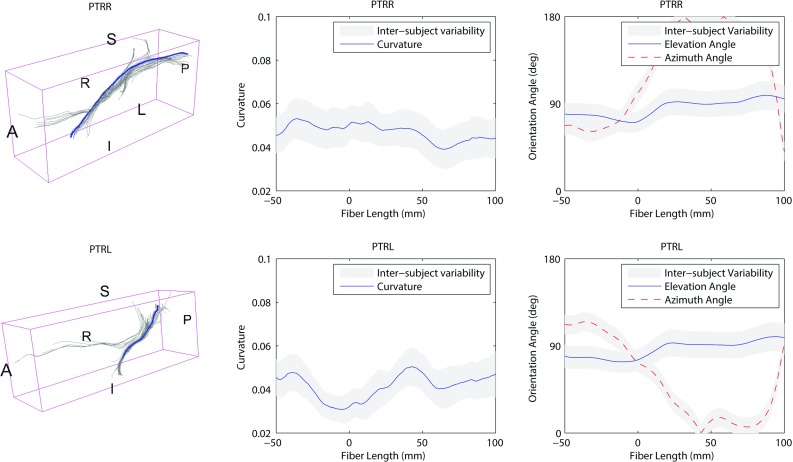
Inter-subject variability of the 3D orientation of the posterior thalamic radiation (PTR). *On the left* evolution of the components of the average tangent unit vector along the reference fiber. *On the right* average curvature along the reference fiber. The origin of the plot corresponds to the center of ROI

## Appendix 1

The following appendix reports detail about the FE model validation.

The KTH aniosotropic model has been extensively compared to intracranial pressure experiments (Giordano and Kleiven 2016), relative skull-brain motion experiments (Giordano and Kleiven 2014; Giordano et al. 2014; Giordano and Kleiven 2016) and brain deformation experiments (Giordano and Kleiven 2014, 2016). The experimental data were taken from Hardy et al. (2001, 2007). In these experiments, the intracranial pressure were measured by implanting transducers at the coup and countercoup location, while the motion between the skull and the brain was measured using a high-speed biplane X-ray and polystyrene neutral density targets (NDTs). The NDTs were implanted into the occipitoparietal and temporoparietal regions of the brain and the cadaver head was suspended in a fixture that permitted rotation and translation. Following, experimental results from impacts C755-T5 and C383-T1 are reported (Figs. 19, 20).

## Appendix 2

The following appendix reports detailed plots of the inter-subject variability of the white matter tract 3D orientation. Fibers were seeded from the medial lemniscus (ML), anterior, superior, and posterior corona radiata (ACR, SCR, PCR) and posterior thalamic radiation (PTR) (Figs. 21, 22, 23, 24, 25).
